# Hyperosmotic cold shock mouse melanoma cells encapsulated with doxorubicin for targeted treatment of melanoma

**DOI:** 10.3389/fonc.2024.1403719

**Published:** 2024-05-01

**Authors:** Weihui Kong, Chengran Wang, Hui Wang, Haiou Liu, Jianhui Mu, Jinlan Jiang, Congxiao Zhang

**Affiliations:** ^1^ Department of Stomatology, The First Hospital of Jilin University, Changchun, China; ^2^ Scientific Research Center, China-Japan Union Hospital, Jilin University, Changchun, China; ^3^ Spine Surgery, China-Japan Union Hospital, Jilin University, Changchun, China

**Keywords:** hyperosmotic cold shock, tumor microenvironment, chemotherapy, targeting, melanoma

## Abstract

**Background:**

The primary treatment strategies for melanoma include surgical excision, chemotherapy, and radiotherapy. However, the efficacy of these treatments is often limited by drug resistance, recurrence, and severe side effects. Therefore, we aimed to develop a targeted drug delivery system capable of selectively locating tumor sites to minimize systemic toxicity and enhance therapeutic efficacy. This cell drug delivery system can also deliver chemotherapeutic drugs to the tumor microenvironment.

**Methods:**

We treated B16F10 cells with hyperosmotic cold shock (HCS) to obtain and characterize HCS cells. We then investigated the anti-tumor effects and immune activation capabilities of these cells and explored their potential as a targeted drug delivery system.

**Results:**

HCS cells not only maintained an intact cellular structure and tumor antigens but also exhibited high expression of the homologous melanoma-associated antigen glycoprotein 100. These cells demonstrated an exceptional capacity for loading and releasing doxorubicin, which has chemotherapeutic anti-tumor effects. HCS cells can precisely target the tumor microenvironment to minimize systemic toxicity, inducing an immune response by activating CD3+ and CD4+ T cells.

**Conclusion:**

HCS cells are non-carcinogenic, with both cellular and tumor antigens intact; thus, they are suitable drug delivery carriers. Our findings highlight the potential of HCS cells for carrying doxorubicin because of their high drug-loading efficiency, effective tumor-targeting and anti-tumor effects. Therefore, our results will facilitate the development of melanoma treatments that have higher efficacy than those in the literature.

## 1 Introduction

The incidence of melanoma, a malignant tumor originating from melanocytes, is increasing globally, particularly in Caucasian populations. Owing to its high invasiveness and tendency toward distant metastasis, this disease has a consistently high mortality rate (1). Although early-stage melanoma has a relatively high cure rate through surgical intervention, once metastasis occurs, the prognosis of patients typically deteriorates rapidly, significantly shortening the median survival period (2, 3).

At present, the primary treatment strategies for melanoma include surgical excision, chemotherapy, radiotherapy, photothermal therapy, and immunotherapy (4). Surgical intervention is the preferred treatment for patients with early-stage melanoma (5); however, its efficacy against advanced or metastatic melanoma is limited (6). Although chemotherapy and radiotherapy can extend patient survival to some extent, the duration of their effects is often short and can result in adverse reactions. Photothermal therapy uses light-sensitive agents activated by specific wavelengths of light to generate heat and kill cancer cells. Different from traditional surgery, photothermal therapy is typically noninvasive or minimally invasive, causing less harm to the patient’s body than traditional surgery (7, 8). Photothermal therapy can be combined with other treatment methods, such as chemotherapy and radiotherapy, to enhance therapeutic outcomes (9–14). However, its effectiveness is subject to certain limitations, such as tissue penetration and dependence on photosensitizers. Despite the target of photothermal therapy being tumor cells, the surrounding normal tissues may be susceptible to heat damage. Additionally, the cost of photothermal therapy equipment and materials is relatively high, decreasing availability.

Advances in immunotherapy, especially the application of immune checkpoint inhibitors, are promising in the treatment of advanced melanoma. Immunotherapeutic drugs activate the immune system to combat cancer and are highly efficacious in the treatment of advanced melanoma (15, 16). However, immunotherapy has limitations, including partial patient resistance to treatment, few effective biomarkers to predict treatment response, and the precise selection of patients suitable for immunotherapy (17, 18). Cells have been considered promising drug delivery systems because of their high biocompatibility and low immunogenicity, allowing them to avoid removal by the reticuloendothelial system (19). Additionally, cells can communicate with the cells of tissues or organs in the body, allowing them to accumulate in specific tissues or organs to deliver targeted drugs (19–21). However, clinical treatments that use living cells have reported challenges, such as the rapid loss of cellular activity outside the body and the low drug capacity of many living cells. Aiming to overcome these limitations, researchers have developed technologies based on freezing biological organisms to inactivate living cells (22, 23).

To improve the storage time, drug-carrying capacity, and targeting ability of cell carriers and to reduce the cytotoxicity of chemotherapeutic agents, we prepared therapeutically inactivated tumor cells by using high permeability cryopreservation technology. These cells can eradicate the growth potential and tumor-forming capabilities of cancer cells while maintaining the complete cellular structure and the full range of tumor antigens. Importantly, hyperosmotic cold shock (HCS) cells exhibit elevated levels of the homologous melanoma-associated antigen glycoprotein 100 (gp100), enabling effective delivery of doxorubicin (DOX) to the tumor microenvironment. Additionally, HCS cells loaded with DOX can induce an immune response by activating CD3+ and CD4+ T cells, inhibiting tumor growth. The combination of the targeted specificity of HCS cells, immune response, and chemotherapeutic effect of DOX has demonstrated substantial anti-tumor efficacy in animal models.

## 2 Materials and methods

### 2.1 Materials

DOX and Cyanine 5.5 (Cy5.5, C41H44N2O14S4) were purchased from Yuanye (Shanghai, China). Fluorescein isothiocyanate/propidium iodide (PI)/calcein AM, 3, 3′-dioctadecyloxacarbocyanine perchlorate (DiO), BCA protein assay kits, and RIPA lysis buffer were purchased from Beyotime Biotechnology (Shanghai, China). Phosphate-buffered saline (PBS), penicillin-streptomycin, fetal bovine serum, and high-glucose Dulbecco’s modified Eagle’s medium were purchased from Thermo Fisher Scientific (Waltham, MA, USA). 4’, 6-diamidino-2-phenylindole (DAPI), phalloidin, phenylmethanesulfonyl fluoride, and Cell Counting kit-8 (CCK-8) were purchased from Service Bio (Wuhan, China). Caspase 9 antibody, BAX antibody, Ki67 antibody, CD3 antibody, CD4 antibody, and β actin antibody were purchased from Proteintech Company (Wuhan, China). Gp100 was purchased from Affinity Biosciences (Santa Clara, CA, USA). B16 cell lines were donated by the National Engineering Laboratory for AIDS Vaccines at Jilin University (24), and the use of these cell lines received approval from the institutional review board for research ethics at the China-Japan Union Hospital of Jilin University.

### 2.2 Preparation of HCS cells

The preparation process was based on previously reported methods (19, 23, 25) with minor modifications. Initially, B16F10 cells were harvested, centrifuged at 1000 rpm for 5 min, and resuspended in 40% glucose. Subsequently, the cells were placed in a 4°C refrigerator for 1 h, followed by centrifugation at 1700 rpm for 5 min to discard the supernatant. The cells were then directly placed into cryovials and stored in a −80°C freezer without controlling the cooling rate. The cells were stored overnight at −80°C and thawed at 37°C before centrifuging at 1700 rpm for 5 min. After a single PBS wash, HCS cells were stored in PBS at 4°C.

### 2.3 Characterization of HCS cells

For scanning electron microscopy, the cells were fixed in an electron microscope fixative for 30 min. After rinsing thrice with PBS, the cells were dehydrated with graded ethanol. After critical point drying, a thin layer of gold was sputtered onto the samples, followed by scanning electron microscopy.

For cell size analysis, an image containing approximately 100 cells was captured using a confocal laser scanning microscope, and cell size was measured using ImageJ software (26). HCS cell viability was analyzed using fluorescence staining with calcein AM and PI. In brief, 1 × 10^6^ HCS or B16 cells were suspended in 1 mL PBS. PI was added, and the cells were stained at room temperature (21°C-25°C) for 5 min. After centrifugation (1000 rpm for 3 min) and washing with PBS, calcein AM/PI detection solution was added, and the cells were incubated at 37°C in the dark for 30 min. After staining, the samples were observed under a laser confocal microscope (Olympus, JP).

Fluorescence staining with F-actin-bound phalloidin and DAPI was performed to observe the cellular cytoskeleton. Cells were cultured on coverslips, the culture medium was removed, and the cells were washed twice with pre-warmed PBS at 37°C. After centrifugation at 1000 rpm for 5 min, cells were covered with 4% paraformaldehyde and incubated at room temperature for 30 min. Subsequently, the cells were washed with PBS two or three times for 10 min each and then permeabilized with Triton X-100 (0.1%) at room temperature for 5 min. After another two or three washes with PBS for 10 min each, the cells were completely covered with freshly prepared phalloidin staining working solution and incubated in the dark for 1 h. The cells were then washed with PBS two or three times for 5 min each, followed by the addition of DAPI staining solution to cover the cells for nuclear staining for 5 min. Next, the cells were washed with PBS two or three times for 1 min each.

The protein preservation status of HCS cells was analyzed using Coomassie Brilliant Blue staining. The expression level of gp100 was analyzed using western blot.

### 2.4 Viability and proliferation of HCS cells

For the in vitro proliferation of HCS and B16F10 live cells, both cell types were seeded into a 96-well plate at a density of 8 × 10^3^ cells per well. After 24, 48, and 72 h of incubation, the complete culture medium containing 10% CCK-8 solution was added to each well. After 2 h of incubation, the absorbance was measured at 450 nm.

### 2.5 Drug loading of HCS cells

To prepare drug-loaded HCS cells (HCS-DOX), after washing, the HCS cells were resuspended in DOX solution. After 24 h of incubation, HCS-DOX were obtained using centrifugation at 1700 rpm for 5 min, and the particles constituted HCS-DOX. The DOX concentration in HCS-DOX was measured using high-performance liquid chromatography (HPLC). The loading of DOX into HCS cells was verified based on the zeta potential. DOX loading was confirmed through confocal microscopy, using DiO for cell membrane staining and DAPI for nuclear staining.

### 2.6 Drug release efficiency of HCS-DOX

We investigated the release efficiency of DOX by using dialysis bags at pH 7.4 and 5.5 to mimic the tumor microenvironment. We placed dialysis bags containing 1 mL DOX-HCS solution into 50 mL centrifuge tubes and added 10 mL PBS at pH 5.5 or 7.4 as release media. These setups were then placed on a shaker at 37.5°C. At 2, 4, 8, 12, and 24 h, equal volumes of the release medium were sampled, and the released DOX content was determined using HPLC.

### 2.7 In vitro cytotoxicity assay of HCS-DOX

To assess the cytotoxicity of HCS-DOX in B16 cells in vitro, we used the CCK-8 (Dojindo, Japan) cell viability assay. Initially, B16 cells in the logarithmic growth phase were collected, digested, and centrifuged (1,000 rpm, 5 min, 23°C). Subsequently, the cell concentration was adjusted to ensure that each well (100 μL) contained 10,000 cells. A 96-well plate was then seeded, with 100 μL cell suspension dispensed into each well, and the perimeter wells were filled with sterile PBS. The plate was placed in a standard cell culture incubator at 37°C, with saturated humidity and 5% carbon dioxide, and incubated for 24 h. Upon reaching 80% confluence, the cells were exposed to complete culture media containing varying concentrations of DOX and HCS-DOX and further incubated for 24, 48, or 72 h. Each group comprised three parallel controls. After discarding the drug-containing media, 100 μL fresh complete culture medium mixed with CCK-8 solution was added to each well and incubated for an additional 2 h. Finally, absorbance was measured at 450 nm by using a microplate reader (Tecan, Swit).

### 2.8 In vitro cell apoptosis assay of HCS-DOX

To investigate the apoptotic effects of HCS-DOX on malignant melanoma cells in vitro, we used flow cytometry and western blot analyses for quantitative assessment. B16 cells were plated in a six-well plate at a density of 8 × 10^5^ cells/well and incubated overnight in Dulbecco’s modified Eagle’s medium containing 10% fetal bovine serum. PBS and HCS cells, DOX (12.5 μg), and HCS-DOX (HCS cells containing 12.5 μg DOX) were added to respective wells. After incubation for 6 h, the cells were centrifuged (1,000 rpm, 5 min, 23°C) in EDTA without trypsin and collected. The cells were stained using an annexin V-fluorescein isothiocyanate/PI apoptosis detection kit and incubated in darkness, followed by the identification of apoptotic cells through flow cytometry. Western blot analysis was conducted to assess the protein levels of cleaved caspase-9 and BAX.

### 2.9 In vivo anti-tumor effect of HCS-DOX

Female C57BL/6J mice aged 6–8 weeks (supplier: Beijing Huafukang Biological Technology Co., Ltd., China) were used. All animal procedures strictly followed the guidelines outlined in the “Guide for the Care and Use of Laboratory Animals” of the National Institutes of Health, and approval was obtained from the Animal Care and Use Committee of Jilin University, China.

In the first study, a B16 melanoma model was established through the subcutaneous injection of 1 × 10^6^ B16 cells (9). Mice carrying tumors (6–8 weeks old) were then divided into four groups (n = 6 per group): PBS, HCS cells, DOX, and HCS-DOX at a DOX dose of 3 mg/kg. Drug administration began 7 d after tumor implantation (designated as day 0), followed by intravenous injection on days 0, 3, 6, 9, and 12. Starting from day 0, we measured the body weight every 2 d and tumor volume every 3 d. Tumor volume (V) was calculated using the formula V = (length) × (width)^2^/2, where the length and width represent the maximum and minimum tumor diameters, respectively (27). Seven days after the last administration, the mice were euthanized, and tissues, namely tumors and major organs, were collected. The tissues were preserved using 4% paraformaldehyde for hematoxylin and eosin (H&E) staining and immunohistochemistry.

In another study, a B16 melanoma model was established by subcutaneously injecting 1 × 10^6^ B16 cells into mice. When the average tumor size reached 100 mm^3^, the mice were divided into negative controls (n = 3, treated with physiological saline), positive controls (n = 3, treated with Cy5.5), and the Cy5.5-labeled HCS cells group (n = 3) (28). Each animal received a single intravenous injection, and live imaging was conducted at different time points (1, 2, 3, 4, 5, 6, 7, and 24 h) by using a small animal imaging system with the same acquisition time and filter settings. The fluorescence intensity of the tumors was quantified from the obtained images, and the tumor retention percentage was determined.

### 2.10 Statistical analyses

All experimental results are presented as the mean ± standard deviation. One-way analysis of variance was employed for data analysis. An unpaired t test was used to evaluate the significance of differences between groups. A P value of less than 0.05 was deemed significant. All statistical evaluations were conducted using GraphPad Prism software (version 9.0.0).

## 3 Results

### 3.1 Preparation and characterization of HCS cells

**Figure 1:**
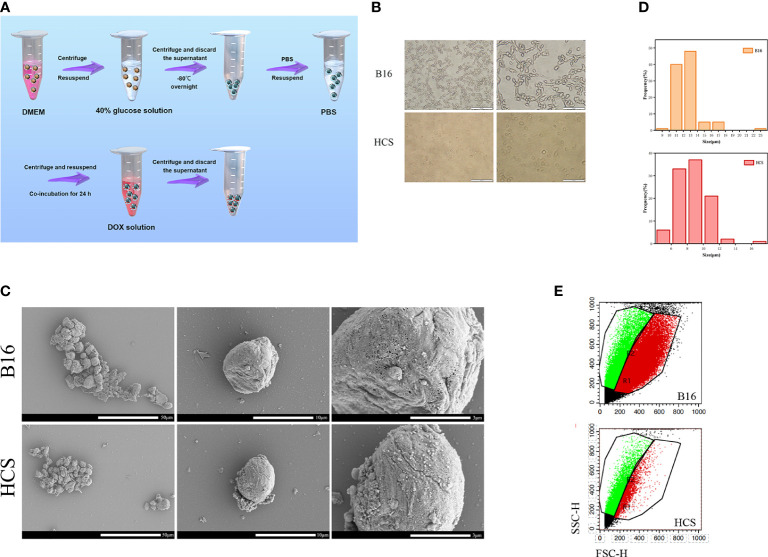
Preparation and characterization of HCS cells. **(A)** Scheme showing the procedure to prepare HCS-DOX. **(B)** Cell morphology of B16 and HCS cells under light microscope. Scale bars are 200 and 100 µm, respectively. **(C)** Scanning electron micrographs of B16 and HCS cells. Scale bars are 50, 10, and 3 µm, respectively. **(D)** Sizes of B16 and HCS cells. Cells were imaged using confocal microscopy; cellular size was measured using ImageJ (cell numbers = 100). **(E)** Flow cytometry analysis of B16 and HCS cells, respectively, under the same voltages.

HCS cells and HCS-DOX were prepared (**Figure 1A**). The morphology of B16F10 and HCS cells under an optical microscope is shown in **Figure 1B**. Scanning electron micrographs indicated that compared with normal mouse skin melanoma cells (B16F10), HCS cells maintained normal cellular morphology with increased surface roughness (**Figure 1C**). The average size of HCS cells (8.7 μm) moderately decreased (**Figure 1D**), consistent with the forward scatter data captured using flow cytometry. Side scatter information from flow cytometry indicated a similar internal complexity between B16F10 and HCS cells (**Figure 1E**).

**Figure 2:**
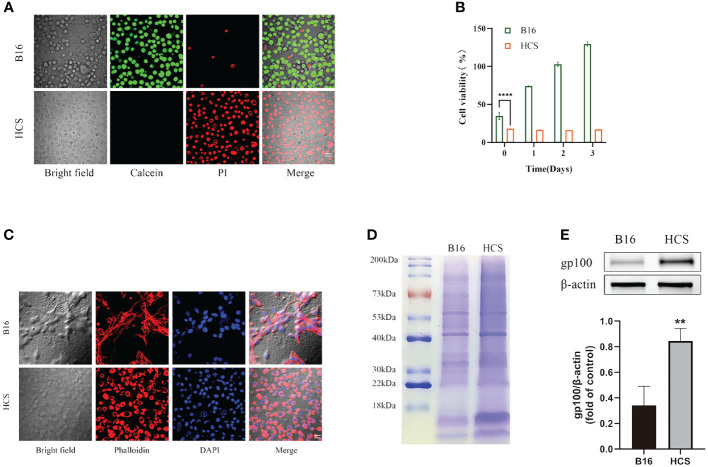
Preparation and characterization of HCS cells. **(A)** Cell death analysis of B16 and HCS cells by Calcein AM/propidium iodide (PI) staining. **(B)** Cell viability analysis of B16 and HCS cells, respectively, by CCK-8 assay. **(C)** Analysis of F-actin distribution of B16 and HCS cells by Phalloidin/4’, 6-Diamidino-2-phenylindole (DAPI) staining. **(D)** SDS-PAGE analysis of B16 and HCS cells. Both samples were concentrated to ensure distinct protein bands. **(E)** Representative western blot of glycoprotein 100 (gp100) in B16 and HCS cells. Quantitative analysis of western blot for gp100. Bars represent means ± SD (n = 3). P values < 0.05 were considered to be significant (**P < 0.01; ****P < 0.0001).

The confocal laser scanning microscopy results showed that almost all HCS cells were stained with PI, indicating that they were dead in contrast with the negligible PI fluorescence in B16F10 cells (**Figure 2A**). Thus, compared with B16F10 cells, HCS cells did not exhibit any proliferative capacity, as shown by the CCK-8 assay (**Figure 2B**).

To determine the functional characteristics of the prepared HCS cells, we conducted phalloidin staining analysis of the cytoskeleton of B16F10 and HCS cells. Compared with that from the live cells, the fluorescence signals from cytoskeleton staining in HCS cells indicated preservation of the cellular structure (**Figure 2C**). Additionally, we analyzed the protein composition of B16F10 and HCS cells by using gel electrophoresis. The results showed that HCS cells had a protein profile similar to that of B16F10 cells, suggesting that most proteins expressed in live cells were retained in HCS cells and that the protein expression in HCS cells was stronger than that in B16F10 cells. Gp100 was upregulated in HCS cells (**Figure 2E**).

### 3.2 HCS cells as drug carriers

**Figure 3:**
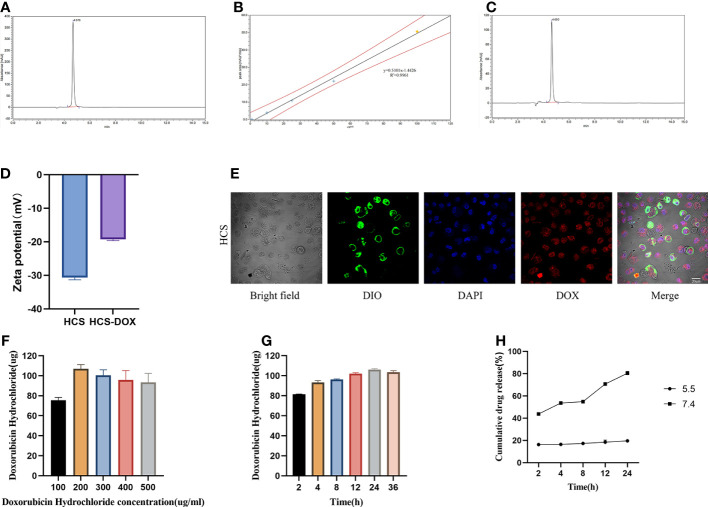
HCS cells as drug carriers. **(A)** Determination of DOX standard through high-performance liquid chromatography (HPLC). **(B)** Standard curve for DOX concentration. **(C)** Determination of DOX in HCS-DOX by HPLC. **(D)** Zeta potentials of HCS cells and HCS-DOX. **(E)** HCS-DOX was incubated with 3, 3′-dioctadecyloxacarbocyanine perchlorate (DiO) and DAPI and then imaged using confocal laser scanning microscopy. Scale bar = 20 μm. DOX loading at **(F)** different concentrations and **(G)** under different conditions. **(H)** Release profile of DOX from HCS-DOX. Data are expressed as mean ± SD (n = 3).

We constructed a cell drug delivery system (DOX-HCS) by loading DOX into HCS cells through co-incubation, followed by determining the drug loading amount of DOX in DOX-HCS by using HPLC. The HPLC chromatogram of the DOX standard sample showed a retention time of approximately 4.7 min (**Figure 3A**). The standard curve equation was as follows: Y (peak area) = 0. 5101X (DOX concentration) - 1.4426 with an R^2^ value of 0.9961 (**Figure 3B**). Subsequently, we measured the amount of DOX loaded into the DOX-HCS, and its retention time was the same as that of the DOX standard (**Figure 3C**), indicating the successful preparation of DOX-HCS. The zeta potentials of the HCS cells and DOX-HCS are shown in **Figure 3D**, confirming the successful loading of DOX into HCS cells. Additionally, we validated DOX loading using cell membrane DiO and nuclear DAPI staining (**Figure 3E**).

Next, we investigated the DOX loading capacity of HCS cells after treatment with different DOX concentrations for varying durations. A total of 1 × 10^6^ HCS cells were co-incubated with DOX at 4°C, and the DOX content in DOX-HCS was detected using HPLC. As shown in **Figure 3G**, the results demonstrate a positive correlation between incubation time and DOX loading amount, with the highest loading observed at 24 h. Further extension of the incubation time to 36 h did not result in additional increases in the loading amount. Additionally, the loading amount increased with the elevation of DOX concentration, peaking at a concentration of 200 μg/mL, beyond which the loading amount ceased to increase (**Figure 3F**). Therefore, using a DOX concentration of 200 μg/mL, the highest loading capacity was achieved after 24 h of incubation, with a loading amount of 106.073 μg per 1 × 10^6^ HCS cells, surpassing a loading efficiency of 50%.

We determined the DOX release profile of the DOX-HCS system. The results revealed that at pH 5.5, approximately 43.8% of DOX was rapidly released from the DOX-HCS within the initial 2 h. Subsequently, the release rate slowed until approximately 80.4% of DOX was released within 24 h. At pH 7.4, approximately 16.3% of DOX was released from DOX-HCS within the first 2 h. After 24 h, the released DOX amounted to approximately 19.6% (**Figure 3H**).

### 3.3 In vitro anti-tumor effects of HCS-DOX

**Figure 4:**
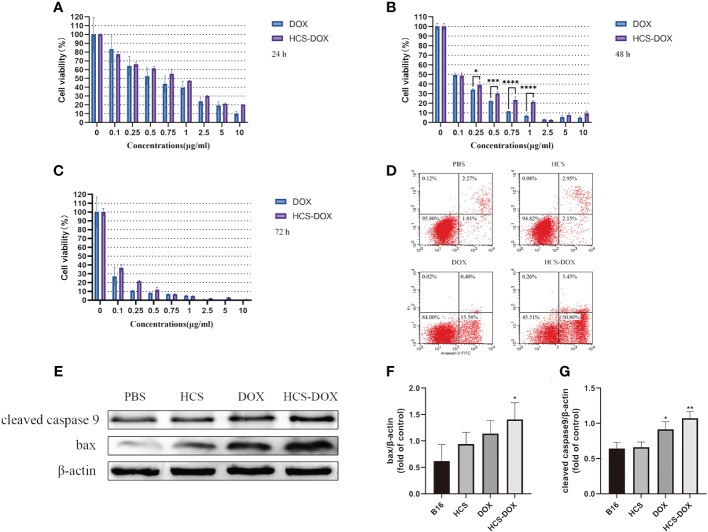
In vitro anti-tumor effects of HCS-DOX Cell viability of B16 cells incubated with various concentrations of free DOX and HCS-DOX at **(A)** 24, **(B)** 48, and **(C)** 72 h. **(D)** PBS, HCS cells, DOX, and HCS-DOX-treated B16 cells were stained with annexin V and PI for FACS analysis. **(E)** Cleaved caspase-9 and BAX protein levels were estimated using western blot analysis. **(F)** Quantitative analysis of western blot for BAX. **(G)** Quantitative analysis of western blot for cleaved caspase 9. Bars represent means ± SD (n = 3). P values ≤0.05 were considered to be significant (*P < 0.05, **P < 0.01, ***P < 0.001, ****P < 0.0001).

We compared the tumor inhibitory effects of free DOX and HCS-DOX at the same drug concentration by using CCK-8 cell viability assays; the results are shown in **Figures 4A–C**. The anti-tumor activity of free DOX and HCS-DOX was concentration- and time-dependent and increased with increasing concentration and exposure time. HCS-DOX demonstrated a cytotoxicity similar to that of free DOX, with slightly lower inhibitory effects than the free drug. For HCS-DOX and free DOX with the same drug dose (DOX: 10 μg/mL), the tumor cell survival rates were 20.3% and 9.9% at 24 h, 9.4% and 5.0% at 48 h, and 0.8% and 0.6% at 72 h, respectively.

We assessed apoptosis in B16 cells by using flow cytometry and protein blotting. The results (**Figure 4D**) indicated that the apoptosis rates of B16 cells in the HCS cells and PBS groups were low. Approximately 15.58% of B16 cells treated with free DOX underwent apoptosis, and approximately 50.8% of the B16 cells treated with HCS-DOX underwent apoptosis, indicating a significant difference. Additionally, protein blotting revealed elevated levels of cleaved caspase 9 and increased expression of BAX, confirming the induction of apoptosis (**Figures 4E–G**).

### 3.4 In vivo anti-tumor effects of HCS-DOX

**Figure 5:**
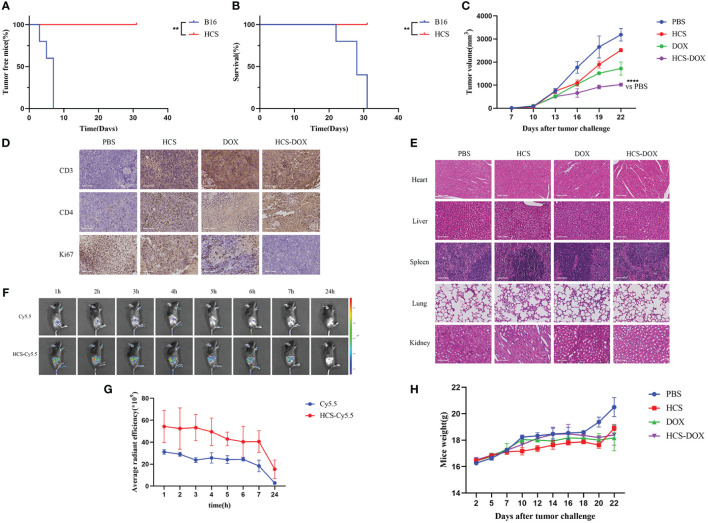
In vivo anti-tumor effects of HCS-DOX. **(A)** Tumor formation and **(B)** overall survival in mice injected with B16 and HCS cells. **(C)** Tumor volumes of melanoma-bearing mice after different treatments. **(D)** Immunohistochemical analysis of Ki67 expression and elevated levels of CD3 and CD4 in tumors of mice in different treatment groups. **(E)** H&E staining of major organs obtained from tumor-bearing mice in different treatment groups. (scale bar = 100 mm). **(F)** Representative images of Cy5.5 and HCS-Cy5.5 at 1, 2, 3, 4, 5, 6, 7, and 24 h. **(G)** Quantitative analysis of fluorescence intensity at different time points. **(H)** Body weights of mice. Data are expressed as mean ± SD. ** indicates P < 0.01, **** indicates P < 0.0001.

Subcutaneous injections of 1 × 10^6^ HCS or B16F10 cells were administered, and the tumor size was recorded every 3 d to observe tumor formation. The results showed the rapid proliferation of B16F10 cells in mice, with tumor formation observed in all mice injected with B16F10 cells within 7 d (**Figure 5A**). By day 31, all mice injected with B16F10 cells had died, and mice injected with HCS cells showed no tumor formation within 31 d, with all mice surviving (**Figure 5B**).

In vitro studies confirmed that the anti-tumor effect of DOX did not diminish when loaded onto HCS cells. Subsequently, we tested the anti-tumor effects of HCS-DOX in vivo. Administration was performed every 3 d, starting from day 0, with measurements of tumor volume and mouse weight collected at each administration. As shown in **Figure 5C**, the tumor volume in the control group rapidly increased after inoculation, and it was partially inhibited after the administration of DOX or HCS-DOX. Compared with the tumor inhibitory effect of DOX treatment, that of HCS-DOX may be enhanced by the targeted action of HCS cells and their immune activation capability.

The experimental results revealed that mice receiving HCS-DOX exhibited the lowest Ki67 expression in tumors. Compared with mice in the other treatment groups, those treated with HCS-DOX showed increased infiltration of CD3+T and CD4+T lymphocytes into the tumor microenvironment (**Figure 5D**).

Next, we assessed the safety of the cellular drug delivery system. The dissolution and rupture of cell nuclei or the disappearance and curling of cell membranes are indicators of cellular necrosis or apoptosis. H&E staining of the hearts, livers, spleens, and kidneys from all groups did not reveal significant changes in the cell membranes or nuclei, that is, no apparent cellular apoptosis or necrosis (**Figure 5E**). Additionally, we used a small animal in vivo imaging system to observe the tumor-targeting efficiency of HCS cells in tumor-bearing mice. The results showed a pronounced Cy5.5 signal at 2 h post-injection (**Figures 5F, G**). Systemic toxicity was monitored by observing changes in the body weight of tumor-bearing mice. The results indicated that compared with the control group’s body weight, the other treatment groups’ body weights did not significantly decrease, and all treatment groups showed a slight increase in body weight at the end of the treatment (**Figure 5H**).

## 4 Discussion

Although the overall incidence of various cancers has a declining trend, the mortality rate of melanoma remains high. The development of immune checkpoint inhibitors such as anti-CTLA-4 and anti-PD-1 antibodies has significantly improved tumor regression and long-term cancer control in patients with melanoma (29, 30). However, the application of immunotherapy has limitations, such as partial patient resistance to treatment (18). Additionally, immune checkpoint inhibitors may have limitations, including novel side effects known as immune-related adverse events, restricted tumor penetration, and inadequate pharmacokinetics (31).

In this study, we successfully cultured HCS cells by using a simple high-osmolarity cryopreservation strategy adapted from previously reported methods. The preparation of these cells is straightforward and can be used for the treatment of melanoma. HCS cells obtained through high-osmolarity cryopreservation maintained morphological features similar to those of active B16F10 cells, albeit slightly smaller in size. Owing to the presence of protein components consistent with the source cells, we posit that HCS cells retain the immunostimulatory capability of tumor cells (**Figure 2D**) (32, 33). Gp100 plays a crucial role in homologous targeting of melanoma-associated antigens (34, 35). The increased expression of gp100 in HCS cells suggests their ability to effectively target melanoma homologously (**Figure 2E**). The tumorigenicity of live cells is a safety concern in the development of whole-tumor cell vaccines. Therefore, the removal of their proliferative capacity before administration or inoculation is crucial. Our in vitro and vivo experiments demonstrated that all B16F10 cells are killed after HCS treatment, eliminating the proliferative and pathogenic capabilities of tumor cells. Therefore, HCS cells can be used as drug carriers for tumor therapy.

Next, we used DOX as the encapsulated chemotherapeutic agent. DOX is a cytotoxic anthracycline antibiotic commonly used as a broad-spectrum antineoplastic agent in cancer chemotherapy (23, 36). In the in vitro anti-tumor experiment of HCS-DOX, the tumor inhibitory effect of HCS-DOX was slightly lower than that of DOX. This discrepancy may be attributed to the relatively low release of DOX in weakly alkaline culture medium. HCS-DOX can more effectively induce tumor cell apoptosis and inhibit tumor cell proliferation than other groups can. The homologous targeting of HCS-DOX to B16 cells and early apoptosis of B16 cells were the main factors for this discrepancy (34, 37). The in vivo experimental results showed that 2 h after injection, HCS cells significantly accumulated at the tumor tissue, demonstrating a tumor-targeting effect. H&E staining indicated that HCS-DOX did not damage the organs of mice. Additionally, during the experiment, there were no changes in the body weight of the mice in any group. These findings further confirmed the low in vivo toxicity, excellent biocompatibility, and biological safety of the HCS-DOX cell drug delivery system. Compared with the other groups, HCS-DOX exhibited the greatest inhibitory effect on tumor growth in B16-bearing mice, significantly prolonging the survival of tumor-bearing mice. The low expression of Ki67 indicated that HCS-DOX can more effectively induce tumor cell apoptosis and inhibit tumor cell proliferation than other groups can. The elevated levels of CD3 and CD4 indicated a close correlation between therapeutic efficacy and the levels of CD3+T and CD4+T lymphocyte infiltration in the tumor after treatment. The HCS-DOX cell drug delivery system synergizes the cytotoxic effects of DOX with its immunostimulatory effects on HCS cells (38–40), enhancing its anti-tumor effect.

**Figure 6:**
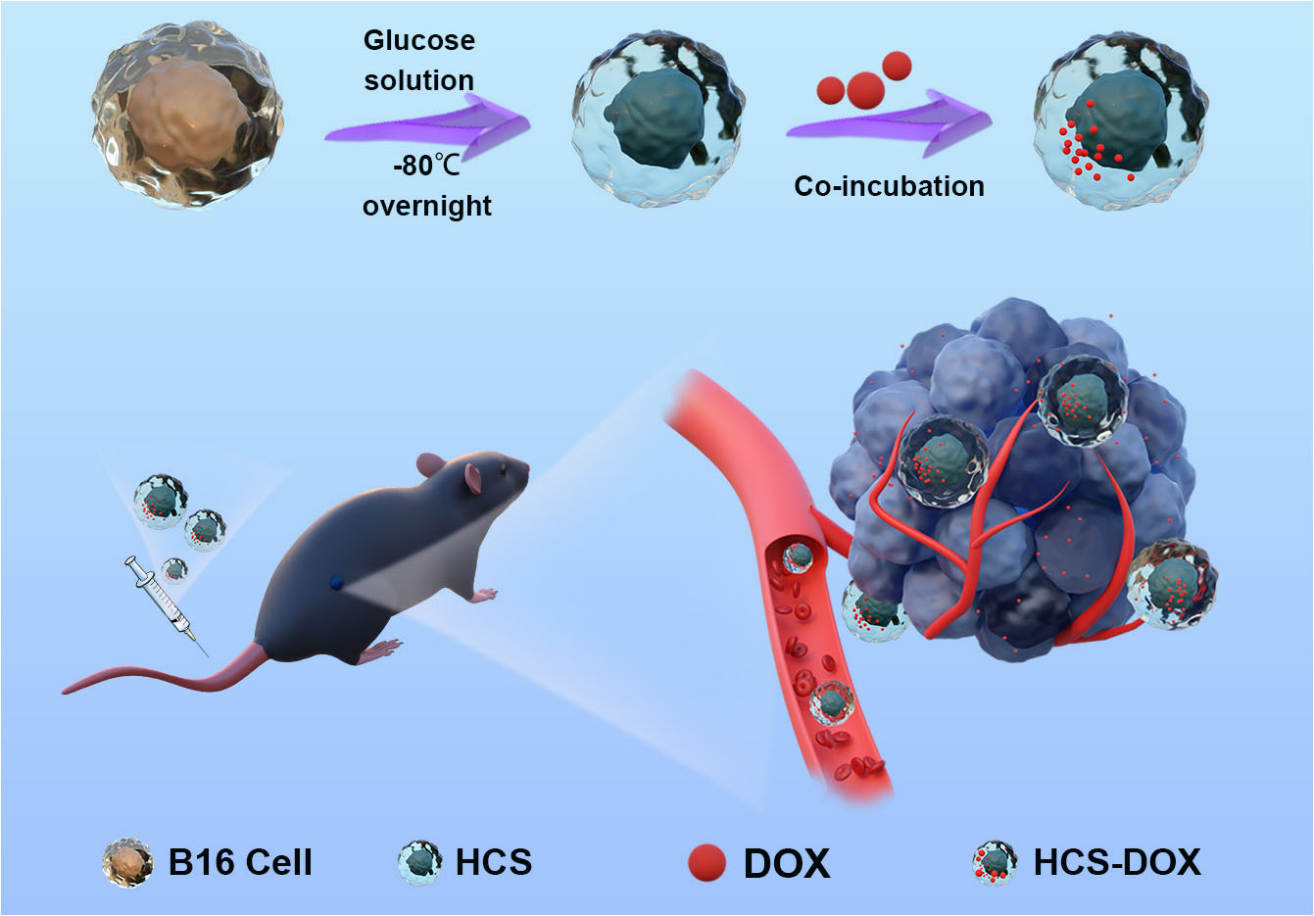
Hypertonic cryo-shocked mouse melanoma cell-encapsulated doxorubicin (DOX)-targeted regimen for the treatment of melanoma. Live B16F10 cells (B16) were first prepared as cell carriers by hypertonic cryo-shock treatment; next, DOX-encapsulated HCS cells (HCS-DOX) were prepared by co-incubating DOX with hyperosmotic cold shock (HCS) mouse melanoma cells for 24 h at 4°C. DOX was efficiently delivered into the tumor microenvironment via hitchhiking HCS cells in the form of HCS-DOX. High targeting efficiency and therapeutic efficacy were demonstrated for the targeted treatment of melanoma models in mice.

Our approach enabled the isolation of tumor cells from patients with melanoma. Using a simple high-osmolarity cryopreservation strategy, we prepared HCS cells with comprehensive tumor antigens. These cells have potential clinical applications and serve as a means of delivering chemotherapeutic drugs to patients (**Figure 6**). This approach holds promise for reducing tumor recurrence rates and post-recurrence tumor volumes. Due to time limitations, this research could not investigate the potential of HCS-DOX to trigger immunogenic cell death in tumor cells and to enhance the immunosuppressive impacts of the tumor microenvironment. Notably, adjuvants or drug combinations, including immunomodulators (e.g., PD-1), can enhance the anti-tumor effects of drugs. Thus, in further research, we plan to use these strategies to further improve the anti-tumor effects.

## Data availability statement

The datasets presented in this study can be found in online repositories. The names of the repository/repositories and accession number(s) can be found in the article/supplementary material.

## Ethics statement

The animal study was approved by the Animal Care and Use Committee of Jilin University, China. The study was conducted in accordance with the local legislation and institutional requirements.

## Author contributions

WK: Writing – review & editing, Data curation, Formal Analysis, Investigation, Project administration, Software, Validation, Writing – original draft. CW: Conceptualization, Methodology, Visualization, Writing – review & editing. CZ: Funding acquisition, Supervision, Writing – review & editing. HW: Data curation, Formal Analysis, Methodology, Project administration, Writing – original draft. HL: Data curation, Formal Analysis, Investigation, Software, Writing – original draft. JM: Investigation, Project administration, Writing – original draft. JJ: Funding acquisition, Resources, Supervision, Writing – review & editing.
